# Understanding the spatial heterogeneity of COVID-19 vaccination uptake in England

**DOI:** 10.1186/s12889-023-15801-w

**Published:** 2023-05-16

**Authors:** Huanfa Chen, Yanjia Cao, Lingru Feng, Qunshan Zhao, José Rafael Verduzco Torres

**Affiliations:** 1 Centre for Advanced Spatial Analysis, University College London, London, UK; 2 Department of Geography, The University of Hong Kong, Hong Kong, China; 3 Chongqing Planning and Design Institute, Chongqing, China; 4 Key Laboratory of Monitoring, Evaluation and Early Warning of Territorial Spatial Planning Implementation, Ministry of Natural Resources, Chongqing, China; 5 Urban Big Data Centre, School of Social & Political Sciences, University of Glasgow, Glasgow, UK; 6 Department of Urban Studies, University of Glasgow, Glasgow, UK

**Keywords:** Spatial accessibility, COVID-19 vaccination, England, MGWR, Socio-economic factors

## Abstract

**Background:**

Mass vaccination has been a key strategy in effectively containing global COVID-19 pandemic that posed unprecedented social and economic challenges to many countries. However, vaccination rates vary across space and socio-economic factors, and are likely to depend on the accessibility to vaccination services, which is under-researched in literature. This study aims to empirically identify the spatially heterogeneous relationship between COVID-19 vaccination rates and socio-economic factors in England.

**Methods:**

We investigated the percentage of over-18 fully vaccinated people at the small-area level across England up to 18 November 2021. We used multiscale geographically weighted regression (MGWR) to model the spatially heterogeneous relationship between vaccination rates and socio-economic determinants, including ethnic, age, economic, and accessibility factors.

**Results:**

This study indicates that the selected MGWR model can explain 83.2% of the total variance of vaccination rates. The variables exhibiting a positive association with vaccination rates in most areas include proportion of population over 40, car ownership, average household income, and spatial accessibility to vaccination. In contrast, population under 40, less deprived population, and black or mixed ethnicity are negatively associated with the vaccination rates.

**Conclusions:**

Our findings indicate the importance of improving the spatial accessibility to vaccinations in developing regions and among specific population groups in order to promote COVID-19 vaccination.

**Supplementary Information:**

The online version contains supplementary material available at 10.1186/s12889-023-15801-w.

## Background

The Coronavirus disease 2019 (COVID-19), caused by the severe acute respiratory syndrome coronavirus-2 (SARS-CoV-2), has been a global pandemic that poses unprecedented health, social, and economic challenges [1]. As of November 2021, the world has confirmed approximately 0.2 billion COVID-19 cases; and in UK alone, over 9 million people have been contracted, with 140,000 deaths. In order to minimise SARS-COV-2 infection and transmissions, public health officials have adopted social distancing as the primary non-pharmaceutical control strategy, until mass vaccination becomes available [2–6].

Vaccine hesitancy has been one of the primary threats to global health, even before the COVID-19 pandemic. The World Health Organisation (WHO) formally defines vaccine hesitancy as ‘delay in acceptance or refusal of vaccine despite the availability of vaccine services’ [7]. Empirical research finds out that the acceptance rates of the COVID-19 vaccines are quite low in most countries, and the lowest acceptance rates were reported in Kuwait, Jordan, Italy, and Russia [8]. The acceptance rate of COVID-19 vaccines differs not only across countries but also within a country. Studies find that the acceptance rate of COVID-19 vaccines is associated with socio-demographic factors [9, 10], including race, age, educational levels, trust in government, among others. In order to address the COVID-19 vaccine hesitancy, it is imperative to understand the spatial patterns of vaccination rates and the role of socioeconomic determinants on vaccination rates.

Existing studies have examined the socio-economic environment and spatial patterns of COVID-19 vaccination rates in different countries, revealing the significant association between socioeconomic determinants and vaccination rates. Soares et al. [11] identified several key socioeconomic factors that were negatively associated with COVID-19 vaccine rates in Portugal, including being younger, loss of income, and having no intention of taking flu vaccine. Benderly et al. [12] found out that older age and higher socioeconomic status were positively associated with vaccination rates in Israel. Agarwal et al. [13] reported that state-level racial disparities in COVID-19 vaccinations in the United States were associated with median income, the disparity in high school education, and political ideology. Nafilyan et al. [14] identified that vaccination rates among elderly adults in England differed considerably across a range of socio-economic characteristics (e.g. ethnicity, religious groups). However, only a linear relationship was captured in these studies. The spatial heterogeneity across the study area was barely discussed in the literature. A US national study by Mollalo & Tatar [15] presented at the county level to examine the spatial relationship between socioeconomic characteristics and COVID-19 vaccination rates. The measurement of spatial accessibility has been applied in many public health domains, such as emergency medical services and primary care services [16, 17]. Several studies in the U.S. have briefly discussed spatial accessibility to COVID-19 vaccination sites in terms of disparities in race/ethnicity and age groups [18, 19]. One study in England evaluated COVID-19 coverage using the average travel time from each neighbourhood [20]. However, quantitative measurement of spatial accessibility was never accounted for as a factor associated with the actual vaccine uptake rates. In addition, the geospatial research on the COVID-19 vaccination uptake in the United Kingdom (UK) is still lacking. More research integrating large-scale and multi-source datasets is needed for a comprehensive picture of the COVID-19 vaccination.

The COVID-19 vaccines have been deployed since the early stage of the pandemic and at scale in the UK. In December 2020, UK regulators issued emergency-use authorisation for COVID-19 vaccines from Pfizer and BioNTech, and later AstraZeneca [21]. In the COVID-19 vaccine delivery plan [22], it is stated that ‘We have always known that vaccines would be our best way out of this pandemic and towards a more normal way of life.’ To maximise the success of the vaccination plan, it is essential to ‘“ensure safe easy access for the whole population’ [22] to the vaccine, which includes spatial accessibility (i.e. the ease of travelling to vaccine sites). However, as discussed, there is a significant lack of the geospatial research on the COVID-19 vaccination accessibility and uptake in the UK. In this study, we reveal the spatial heterogeneity of COVID-19 vaccination uptake and its relationship with socio-economic variables in England.

This study makes contributions to the literature and policy in the following ways: first, it presents the first spatial model of the COVID-19 vaccination rates at the Middle Layer Super Output Areas (MSOA) level in England. Second, in a pioneering move, this study considers the role of spatial accessibility to vaccination sites as a factor influencing vaccine uptake rates, in conjunction with a range of socio-economic variables. Third, the findings can inform public health policymakers to develop tailored strategies to increase uptake of COVID-19 vaccines and adjust local vaccination policy.

## Methods

### Data

**Fig. 1:**
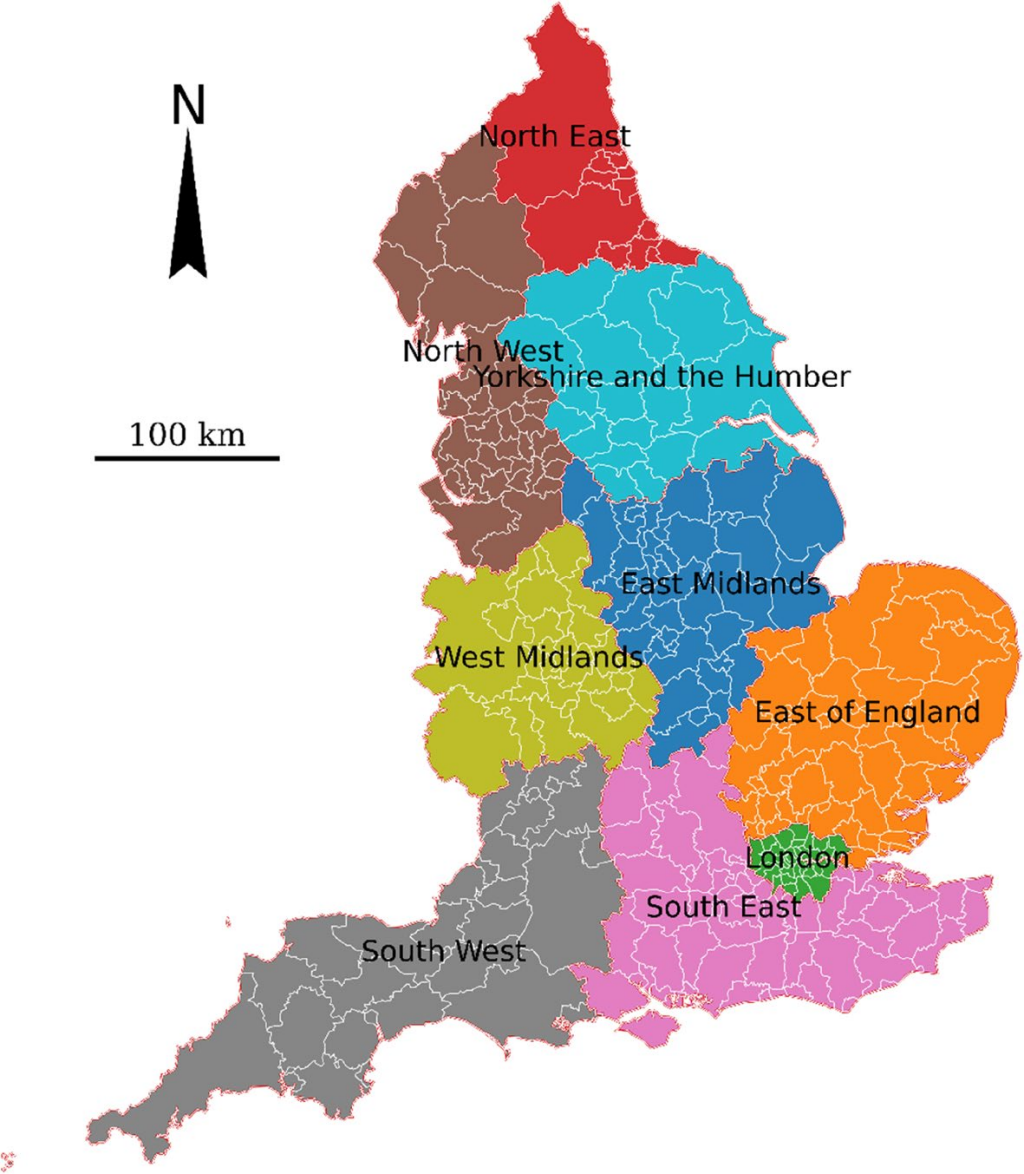
Map of England with the boundary of nine regions (marked by different colours) and local authority districts

Figure 1 shows the map of England, with the boundary of nine regions and local authority districts. In 2020, England has a population of 56.3 million, comprising 84% of the UK population [23]. England was selected as the study area due to its various publicly available data of vaccination sites and uptake as well as socioeconomic variables.

#### Vaccination uptake rates

We collected the MSOA-level weekly number of vaccination uptake by age groups in England from the National Health Services (NHS) England [24], which started on 8 December 2020. According to the vaccine delivery plan [22], NHS has prioritised high-risk residents (including the elderly) and offering vaccines to different age groups in stages. In this study, the dependent variable is the accumulative second-dose vaccination rate among population aged over 18 years as of 18 November 2021, as this date was close to the date when the vaccination site data were retrieved.

#### Demographic data

We used the MSOA from the 2011 UK census as the spatial analytical unit, which is a geographic area designed to improve the reporting of small area statistics in England and Wales. By definition, each MSOA has an average population of 7,500 residents or 4,000 households. England is divided into 6,791 MSOAs.

The most recent MSOA-level demographic information for England is the resident population data from 2019 mid-year estimates [23], which includes the estimated population size by age group and sex. MSOAs are represented by population-weighted centroids based on 2011 census data, which is in line with the demand unit representation in the accessibility study by Luo and Wang [25]. This study uses the population percentage of different age groups and ethnicity from this demographic data (see Table 1).

**Table 1:** The independent variables used in this study

Independent variable	Abbreviation	Definition
Age between 18 and 29%	AGE18_29	Percentage of persons aged between 18 and 29
Age between 30 and 39%^a^	AGE30_39	Percentage of persons aged between 30 and 39
Age between 40 and 49%	AGE40_49	Percentage of persons aged between 40 and 49
Age between 50 and 59%	AGE50_59	Percentage of persons aged between 50 and 59
Age between 60 and 69%	AGE60_69	Percentage of persons aged between 60 and 69
Age between 70 and 79%	AGE70_79	Percentage of persons aged between 70 and 79
Age 80 and older %	AGE_80	Percentage of persons aged 80 and older
White population %^a^	WHITE	Percentage of white ethnicity
Mixed population %	MIXED	Percentage of mixed ethnicity
Black population %	BLACK	Percentage of black ethnicity
Asian population %	ASIAN	Percentage of Asian ethnicity
Other population %	OTHER	Percentage of other ethnicities
Spatial accessibility to vaccination	ACCESS	Spatial accessibility to vaccination computed by E2SFCA
Average household income	INCOME	Average household income
The quintile of Index of Multiple Deprivation	Q_IMD	The quintile of the 2019 English Index of Multiple Deprivation
Car ownership %	CAR	Percentage of households having at least one car or vans

^a^Manually removed from regression models to avoid severe multicollinearity between variables

#### Car ownership

Car ownership (i.e. the proportion of households having at least one car or van) is used as a measure of local travel accessibility [26]. This variable is included as car ownership, as local travel accessibility might exhibit a considerable association with the vaccine uptake rates, especially in rural areas where public transport is not popular.

#### Multiple deprivation index

We used the 2019 English Index of Multiple Deprivation (IMD) [27] as a measure of relative small-area deprivation. The IMD was originally calculated for Lower-layer Super Output Areas in England and then aggregated to the MSOA level. Office for National Statistics (ONS) recommended using the ranks and deciles of IMD rather than the scores as the scores were less easy to interpret. As such, the IMD is represented by an integer variable with values between one (the most deprived) and five (the least deprived), which correspond to the quintile of IMD. The use of IMD quintile is consistent with a recent report on COVID-19 vaccination from ONS [28].

#### Travel duration

The travel duration between each pair of MSOA centroids and vaccination sites is required for computing the spatial accessibility to vaccination. As regions differ considerably regarding travel modes, we used the weighted-mean travel duration that combines driving and public transport, using the regional-level travel mode proportion as the weight. The proportion of driving and public transport in nine regions was derived from [29]. Details of computing the weighted-mean travel duration is available in Additional file 1: Appendix 1.

#### Accessibility to vaccination sites

The addresses of publicly accessible SARS-CoV-2 vaccination sites (*N* = 2,868) in England were retrieved from the NHS England [30] up to 17 November 2021. As the vaccine supply in each site is not available, we assume an equal and unlimited vaccine supply across sites. These sites fall into three types, including vaccination centres (*N* = 108), hospital hubs (*N* = 230), and local vaccination services (*N* = 2530). The local vaccination services include pharmacies and GP-led vaccination services. Although mobile units were in operation as another type of vaccination services, the locations of these units are not publicly available and therefore they were excluded herein. The original vaccination site data contain address and postcode (e.g. ‘Airedale Hospital NHS FT, Skipton Road, Keighley, West Yorkshire, BD20 6TD’), which were geocoded into WGS-84 coordinates of (longitude, latitude) using the Geocoding API from Google Maps Platform [31]. The accessibility to vaccination sites was calculated using the Extended Two-Step Floating Catchment Area, which is specified in the Analyses section. In summary, the independent variables are summarised in Table 1 and will be used in the following analysis.

### Analyses

The following sections will examine the relationship between COVID-19 vaccination uptake rates and a range of socio-economic variables, using three different methods. These models include an ordinary least squares (OLS) method as the baseline, geographically weighted regression (GWR), and multiscale GWR (MGWR). We will calibrate these models and evaluate their accuracy in explaining the variance of COVID-19 vaccination uptake rates in England.

#### Geographically weighted regression

The OLS method fits a global linear model between the dependent and independent variables. When this method is applied in spatial analysis, it is limiting as it assumes that the relationship between dependent and independent variables is spatially homogeneous, which is not true in every spatial context. To relax this assumption, the GWR provides an alternative method to examine the spatial variations in local parameter estimates. More details about GWR is available in Additional file 1: Appendix 2. To our knowledge, GWR has been used in multiple research studies to describe the degree to which socio-economic factors are associated with the COVID-19 morbidity [32–35] and mortality [36], and also COVID-19 vaccination rates [15].

#### Multiscale GWR

To overcome the GWR drawbacks, Fotheringham et al. [37] proposed an extension of the GWR method, MGWR, which computes separate optimum bandwidths for each independent variable. This method improves the ordinary GWR in two aspects: first, it relaxes the assumption that all independent variables influence the response variable within the same spatial scale; second, it alleviates the local multicollinearity problem by minimising the overfitting of GWR and achieves more reliable parameter estimates.

#### Data preprocessing and model evaluation

The dependent and independent variables were transformed to standardised z-score (with zero mean and unit standard deviation) before being used for modelling. The data standardisation serves two purposes: first, it allows for scale-free bandwidths that are comparable across variables; second, it reduces the computational complexity of GWR and MGWR. We used the Python package *mgwr* [38] for building and evaluating the three models.

We evaluated the performance of models using a combination of criteria: Adjusted R^2^ (Adj. R^2^), Akaike Information Criterion (AIC), residual sum of square (RSS), log likelihood, and Moran’s I. A larger adjusted R^2^ is desirable, as it indicates that the model can explain a larger variance of the vaccine uptake rates. On the other hand, a smaller AIC or a smaller RSS is preferred. A smaller AIC implies a more parsimonious model, and a smaller RSS implies that the model explains a larger variance of the vaccine uptake rates (which is similar to a larger adjusted R^2^). In addition, a larger log likelihood means that the model is more likely to be true given the data distribution. Moreover, the Moran’s I statistic is used to test whether the model’s residuals are spatially autocorrelated.

#### Accessibility to vaccination sites

The MSOA-level accessibility to vaccination sites was computed using the Extended Two-step Floating Catchment Area Method (E2SFCA) via the Python library ‘access’ [39]. More details of E2SFCA is available in the Additional file 1: Appendix 3 [40].

## Results

### Variable selection using OLS model

The final OLS model was constructed based on 14 variables, after manually removing two variables to reduce multicollinearity problem. As shown in Table 2, the VIF values for all selected variables were smaller than 10, indicating that multicollinearity is not severe. The OLS residuals were highly clustered across space, as the results of Moran’s I test show that Moran’s I = 0.203, z-score = 27.109, and *p*-value < 0.01 (Table 3). The autocorrelated residuals in OLS violate the independence of errors assumption of the OLS model. Therefore, the estimated coefficients should be interpreted with caution.

**Table 2:** OLS results of COVID-19 vaccination uptake rates in England

Variable	Coefficient	Std.Error	t-Statistic	*p*-value	VIF
Intercept	0.000	0.007	0.000	1.000	NaN
Q_IMD	-0.036	0.015	-2.461	0.014	4.751
AGE18_29	-0.167	0.008	-21.687	0.000	1.338
AGE40_49	0.193	0.017	11.322	0.000	6.566
AGE50_59	0.129	0.009	14.585	0.000	1.776
AGE60_69	0.162	0.016	10.404	0.000	5.503
AGE70_79	0.271	0.021	13.038	0.000	9.745
AGE_80	0.140	0.013	11.063	0.000	3.620
MIXED	-0.126	0.014	-8.766	0.000	4.675
ASIAN	0.037	0.009	4.013	0.000	1.950
BLACK	0.029	0.011	2.562	0.010	2.984
OTHER	-0.126	0.010	-12.315	0.000	2.347
INCOME	0.272	0.013	20.370	0.000	4.022
CAR	0.198	0.015	13.164	0.000	5.089
ACCESS	0.030	0.007	4.093	0.000	1.183

**Table 3:** Comparison of the three models for the vaccination uptake in England

Model		OLS	GWR	MGWR
Evaluation metric	AIC	11,117.141	7981.688	7914.267
	Adj_R2	0.699	0.838	0.832
	RSS	2035.450	910.623	1009.074
	Log-likelihood	-5543.570	-2814.031	-3162.407
	Moran’s_I	0.203	0.029	0.010
	z score	27.109	3.905	1.400
	*p* value	0.000	0.000	0.081

### Model comparison

In comparison with OLS, both the GWR and MGWR achieve better fits with improved adjusted R2, and the local models can explain 84% (from GWR) or 83% (from MGWR) of the variance of the COVID-19 vaccination rates. In terms of AIC, the MGWR is more parsimonious than OLS and GWR. On the other hand, GWR achieves the lowest residual sum of squares (RSS), which is followed by MGWR and OLS. Regarding the spatial distribution of residuals, both OLS and GWR produce a spatially clustered pattern of residuals that is statistically significant (*p* < 0.05), while MGWR produces a random distribution of residuals (*p* > 0.05). This indicates that MGWR effectively mitigates the spatial autocorrelation or clustering of residuals of the COVID-19 vaccination rates.

The bandwidths selected by GWR and MGWR are presented in Table 4 and Table 5. The GWR model obtains a universal bandwidth of 195 (in comparison with a total of 6786 MSOAs in this study). In contrast, the bandwidths selected by the MGWR model vary across variables. Specifically, the bandwidths of the Intercept, AGE18_29, and AGE50_59 are smaller than the GWR bandwidth, indicating that the influence of these variables on the vaccination rates is considerably localised. On the other hand, the bandwidths of several variables are close to the number of spatial units, including BLACK, INCOME, CAR, and ACCESS. That means the relationship between these variables and the vaccination rates is at the global scale. In the following discussion, we will focus on the MGWR model.

**Table 4:** The GWR Results for the parameter estimates

Variable	Mean	STD	Min	Median	Max	Bandwidth
Intercept	-0.166	0.491	-3.888	-0.138	1.373	195
Q_IMD	-0.098	0.174	-1	-0.07	0.276	195
AGE18_29	-0.067	0.139	-0.626	-0.056	0.409	195
AGE40_49	0.306	0.238	-0.467	0.298	1.228	195
AGE50_59	0.14	0.119	-0.216	0.13	0.558	195
AGE60_69	0.169	0.151	-0.31	0.179	0.656	195
AGE70_79	0.327	0.195	-0.395	0.33	0.833	195
AGE_80	0.191	0.148	-0.301	0.179	0.673	195
MIXED	-0.127	0.374	-2.334	-0.065	0.854	195
ASIAN	-0.11	0.535	-3.089	0.012	2.068	195
BLACK	-0.121	1.056	-9.138	-0.041	6.322	195
OTHER	-0.176	0.347	-2.284	-0.144	0.937	195
INCOME	0.186	0.205	-0.551	0.178	0.858	195
CAR	0.271	0.28	-0.732	0.298	0.978	195
ACCESS	0.006	0.15	-0.893	0.033	0.506	195

**Table 5:** The MGWR Results for the parameter estimates using adaptive bandwidth

Variable	Mean	STD	Min	Median	Max	bandwidth
Intercept	0.014	0.284	-0.809	-0.027	1.201	43
Q_IMD	-0.123	0.104	-0.623	-0.103	0.08	233
AGE18_29	-0.079	0.146	-0.589	-0.082	0.405	110
AGE40_49	0.246	0.105	0.086	0.285	0.402	1319
AGE50_59	0.158	0.106	-0.141	0.149	0.433	158
AGE60_69	0.172	0.011	0.147	0.166	0.185	5052
AGE70_79	0.28	0.158	-0.021	0.29	0.582	456
AGE_80	0.196	0.035	0.129	0.187	0.263	1315
MIXED	-0.107	0.033	-0.147	-0.116	-0.07	4334
ASIAN	0.03	0.154	-0.466	0.048	0.384	289
BLACK	-0.05	0.003	-0.054	-0.051	-0.042	6643
OTHER	-0.11	0.081	-0.284	-0.121	0.061	738
INCOME	0.133	0.001	0.13	0.133	0.135	6786
CAR	0.357	0.001	0.354	0.357	0.359	6786
ACCESS	0.026	0	0.026	0.026	0.027	6786

## Discussion

### Model interpretation

In this section, we present and discuss the selected MGWR model results by visualising the parameter estimates that have a statistically significant relationship with the COVID-19 vaccination rates. We focus on interpreting the MGWR model as it is more parsimonious than OLS and GWR and leads to the randomly distributed residuals.

#### Ethnicity

**Fig. 2:**
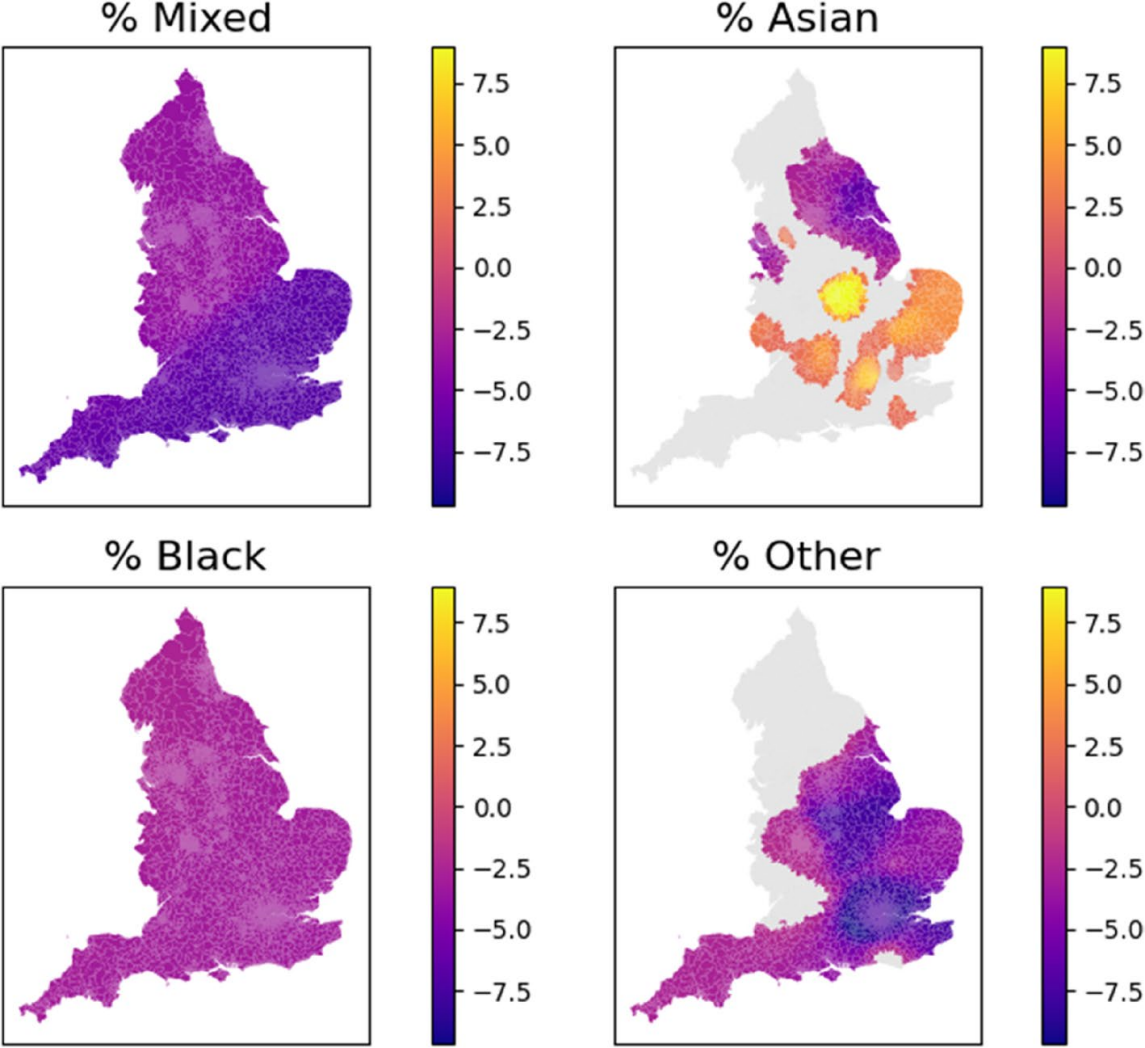
MGWR parameter estimates for the ethnicity proportions. The grey colour pertains to statistical non-significance at 95% interval. The Black, Mixed, and Black ethnicities are associated with a lower vaccination rate across the nation when compared to the White group. The ‘Other’ ethnicity is found to decrease the vaccination rates in the central and southern England, while this relationship is insignificant in most of northern England. The Asian ethnicity is associated with a lower vaccination rate in northeast England but with a higher vaccination rate in the central and southern England

In terms of ethnicity, the White (%) was excluded from the model in order to mitigate multicollinearity between variables. Therefore, the parameter estimate shown in Fig. 2 is the difference of the influence between the given ethnicity and White. Overall, the Mixed and Black ethnicity is associated with a lower vaccination rate across the nation when compared to the White group. The ‘Other’ ethnicity is found to decrease the vaccination rates in the central and southern England, while this relationship is insignificant at the 95% interval in most of northern England. On the other hand, the Asian ethnicity has a negative relationship with the vaccination rates in the Yorkshire and Humber and parts of Northwest, but has a positive relationship with the vaccination rates in the eastern and southern England.

Our findings regarding how ethnicity is related with vaccination are largely consistent with reported vaccination rates (from 8 December 2020 to 15 May 2021) by socio-demographic groups among people over 40 in England [41], although the ethnic classifications are somewhat different. Specifically, this report specified that the ‘White British’ had the highest vaccination rate, followed by ‘Bangladeshi’, ‘Black African’, ‘Black Caribbean’, ‘Chinese’, ‘Indian’, ‘Mixed’, ‘Other’, ‘Pakistani’, and ‘White Other’. Figure 2 finds that most parameter estimates of ‘Black’, ‘Mixed’, and ‘Others’ are negative where these variables are statistically significant, meaning that they are associated with a lower vaccination rates compared with White. The ‘Asian’ group exhibits a spatially heterogeneous relationship with the vaccination rates, showing a negative association in the northeast and positive association in the central and southern England. These results (except Asian) are consistent with the above ONS report that finds the ethnic minorities have a lower vaccination rates than White. This can be attributed to issues of trust in the government or health care system among the ethnic minorities [42]. In addition, while the ONS report presents only an odds of being vaccinated for each ethnicity, this study reveals the nuanced spatial heterogeneity of parameters (e.g. the Asian and Others). These results would facilitate the localised measures for prioritising the ethnic groups with lower tendency of vaccination.

#### Age groups

**Fig. 3:**
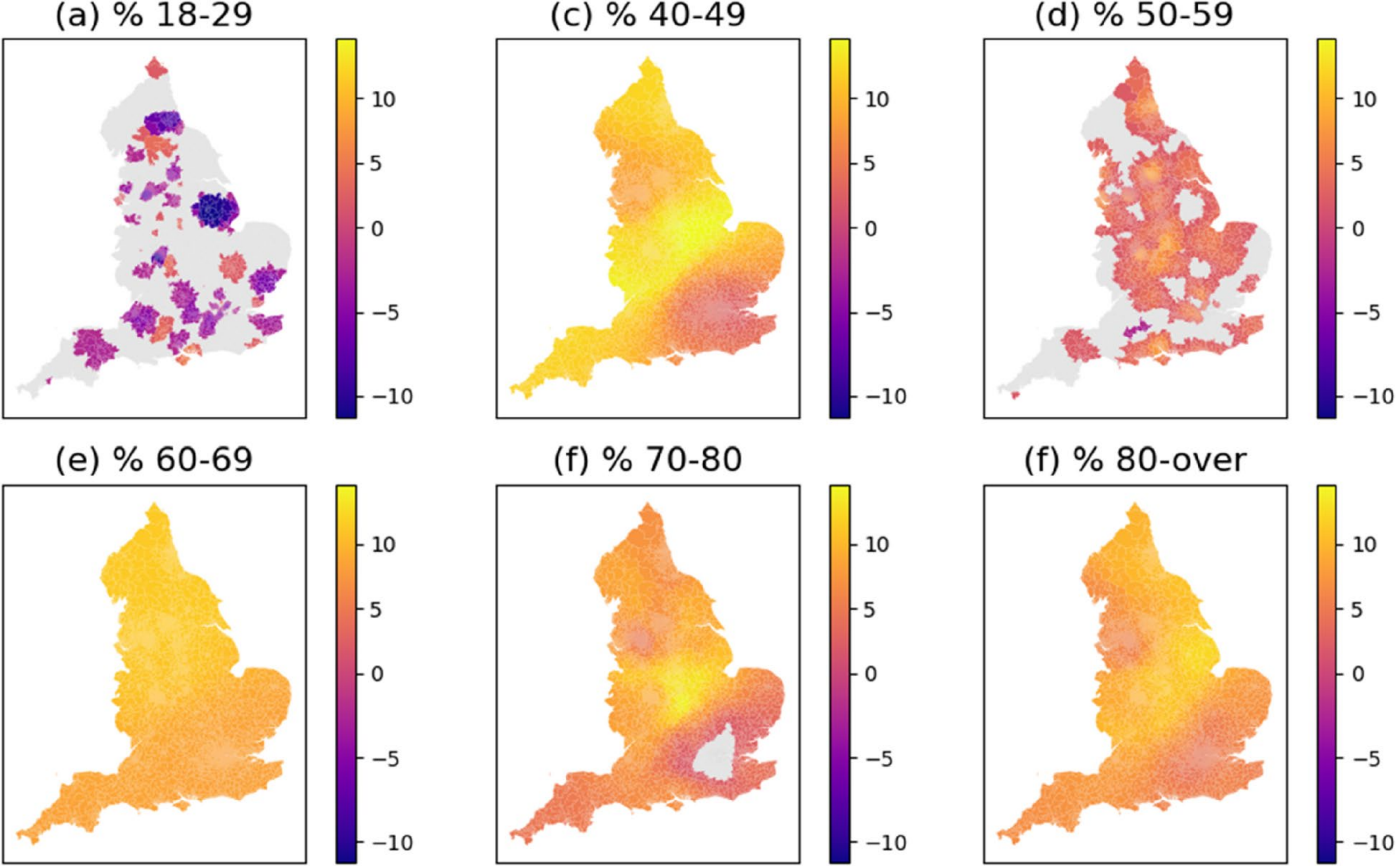
MGWR parameter estimates for the age group variables. The grey colour pertains to statistical non-significance at 95% interval. In comparison with the age 30–39 (the reference age category), the age 18–29 is associated with a lower vaccination rate in several parts of England, implying that young people are less likely to get vaccinated than the 30–39 group. The senior groups (40–49, 50–59, 60–69, 70–79, and 80 over) exhibits a positive association with the vaccination rates, especially in the central and northern England

The parameter estimates of age groups are illustrated in Fig. 3. Note that the group of 30–39 was excluded from the MGWR model and therefore the interpretation of parameter estimates is in comparison with age 30–39. In comparison with the age 30–39, the age 18–29 is associated with a lower vaccination rate in several parts of England, implying that young people are less likely to get vaccinated than the 30–39 group. In contrast, the senior groups (40–49, 50–59, 60–69, 70–79, and 80-over) exhibits a positive association with the vaccination rates, especially in the central and northern England. The results indicate the elderly people are more likely to get vaccinated, which is largely consistent with the ONS study [41] that finds the elderly people has a higher odds of receiving the first dose COVID-19 vaccination.

#### Economic/accessibility/deprivation

**Fig. 4:**
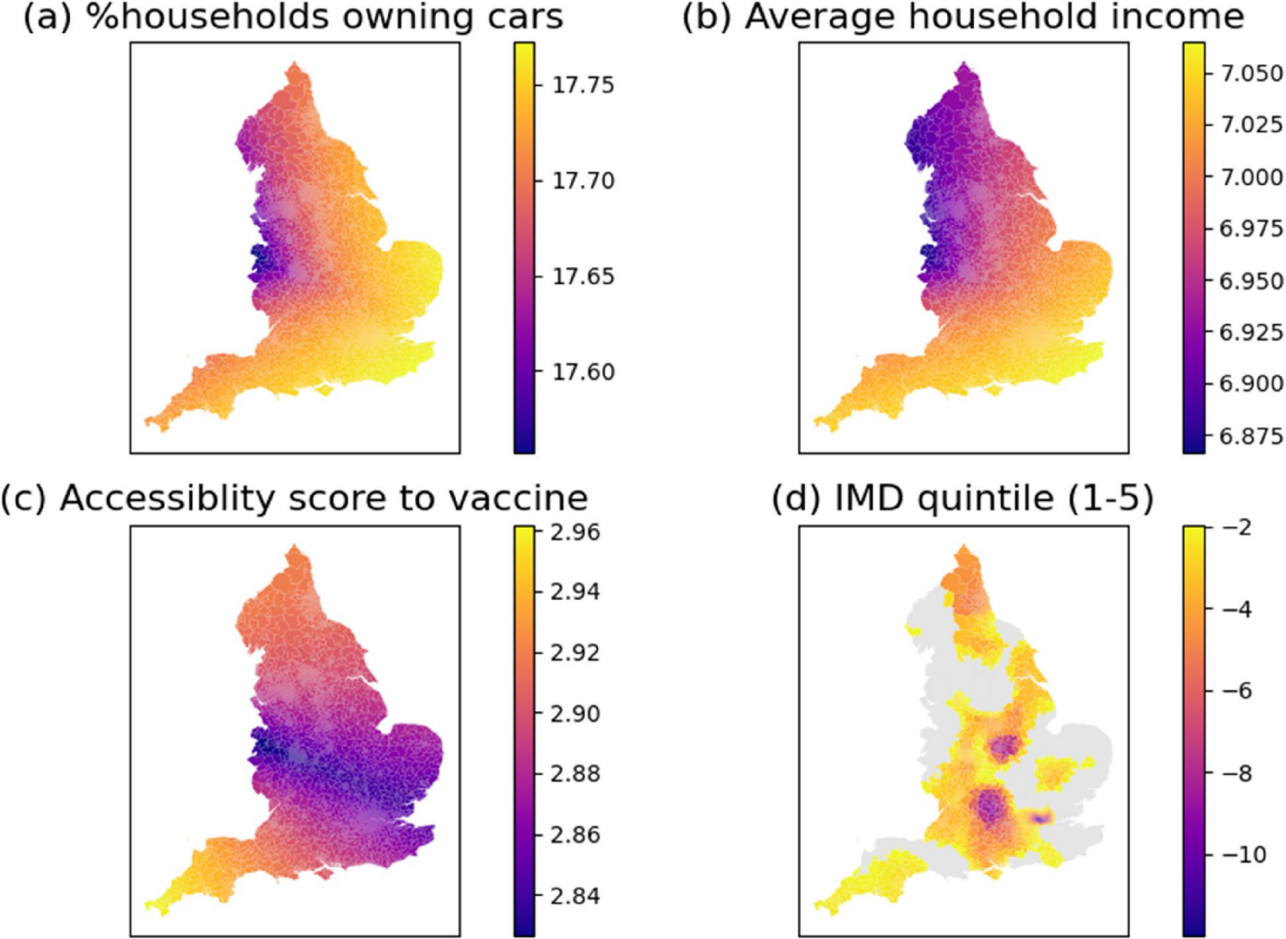
MGWR parameter estimates for various variables. The grey colour pertains to statistical non-significance at 95% interval. Both household car ownership and average household income have a strong and positive relationship with the vaccination rates in most neighbourhoods, indicating that people with better private transport mobility or higher incomes are more likely to get vaccinated. The accessibility measure to vaccination services exhibits a positive association with vaccination rates, although at a less intensive level. IMD quintiles (the first quintile meaning the most deprived) exhibit a negative relationship with vaccination rates in almost half of MSOAs, indicating that the less deprived, the lower vaccination rates

The parameter estimates of the economic, accessibility, and deprivation variables are demonstrated in Fig. 4. As shown, both household car ownership and average household income have a strong and positive relationship with the vaccination rates in most neighbourhoods, indicating that people with better private transport mobility or higher incomes are more likely to get vaccinated. This is aligned with the report by the UK Parliament [43], which points out that accessibility of transport to vaccine facilities is one of the barriers to accessibility of vaccines.

The accessibility measure to vaccination services exhibits a positive association with vaccination rates, although at a less intensive level. This finding confirms the importance of improving the accessibility to vaccination services [22]. In contrast, IMD quintiles (the first quintile meaning the most deprived) exhibit a negative relationship with vaccination rates in almost half of MSOAs, indicating that the less deprived, the lower vaccination rates [42].It can be explained by that people living in more deprived areas have higher rates of most underlying clinical risk factors that enlarge the severity and mortality of COVID-19 and are therefore more likely to get vaccinated [44]. However, this finding contrasts with the ONS report [41], which finds that for the individuals in the ONS Public Health Data Asset dataset, the less deprived, the higher odds of getting vaccinated. The possible explanation of this difference is the different data source and scale: while this study focuses on the MSOA-level vaccine uptake rate, the ONS report uses individual data in the analysis.

### Limitations

This study has several limitations, which has introduced potential bias in the current research and opens avenues for future research. First, other important determinants of vaccine uptake, including education levels and trust in government, were not assessed in this analysis. The absence of these variables might lead to the underfitting of regression models in some areas. Second, the accessibility measurement to the COVID-19 vaccination would be more accurate if the data of the mobile units for vaccination is available. Third, the dynamics of vaccination uptake is not considered in this study. Future research could build spatio-temporal models to simultaneously account for the spatial heterogeneity and evolution of the vaccination uptake, which could inform region-specific and population-group-specific policies in different stages of the mass vaccination.

## Conclusions

In this study, we used multiscale geographically weighted regression models to reveal that the spatial disparity of COVID-19 vaccination uptake rates in England is strongly associated with socio-economic-demographic variables. Moreover, this relationship exhibits considerable spatial heterogeneity and local effects. Overall, the younger groups (18–29, 30–39) are associated with lower vaccination rates than the elderly, whilst higher car ownership or better accessibility to vaccination services lead to higher vaccination uptake rates. On the other hand, the more deprived areas are found to be related to a higher vaccination rate than the less deprived. It is worth noting that the sign and multitude of the relationship likely vary geographically, such as the ‘Asian’ ethnicity and age 50–59.

To our best knowledge, there has been a lack of small-area spatial modelling of COVID-19 vaccination in the UK. For this reason, this quantitative study will serve to support public health management in developing regional-specific policies for maximising vaccination uptake in the ongoing COVID-19 vaccination and other mass vaccination programmes.

## Supplementary Information

**Additional file 1.**

## Data Availability

The data supporting the findings reported in this paper are openly available from the repository of https://github.com/huanfachen/Vaccine_uptake_analysis.

## Abbreviations

AIC: Akaike Information Criterion
COVID-19: Coronavirus disease 2019
E2SFCA: Extended Two-step Floating Catchment Area Method
GWR: Geographically weighted regression
IMD: 2019 English Index of Multiple Deprivation
MGWR: Multiscale geographically weighted regression
MSOA: Middle Layer Super Output Areas
NHS: National Health Services
OLS: Ordinary least squares
ONS: Office for National Statistics
OSRM: Open Source Routing Machine
RSS: Residual sum of square
SARS-CoV-2: Severe acute respiratory syndrome coronavirus-2
UK: United Kingdom
WHO: World Health Organisation
