# Supplementary material for: Understanding the spatial heterogeneity of COVID-19 vaccination uptake in England
Source: BMC Public Health. 2023 May 16;23:895. doi: 10.1186/s12889-023-15801-w (PMC10185460; doi:10.1186/s12889-023-15801-w)
Supplement: Supplementary file 1 — Additional file 1. [file 12889_2023_15801_MOESM1_ESM.docx]

# Supplementary Information

## Appendix 1: calculation of the weighted-mean travel time

The weighted-mean travel duration betwewn MSOAs and vaccination sites was computed as follows. First, the driving duration between each pair of MSOA centroids and vaccination sites was computed by the Open Source Routing Machine (OSRM) [1]. OSRM is a high-performance and open-source routing engine, which is designed to retrieve and apply OpenStreetMap data via HTTP API and other interfaces. OSRM provides varying services including identifying the fastest route between coordinates and computing the pairwise shortest travel times between supplied coordinates. Second, the duration of public transport journeys was computed using the R5R software [2] for R programming language (version 4.1.2) [3]. This uses as its main input OpenStreetMap data to account for the pedestrian network and the timetables for the main public transport modes. The timetables were accessed from the Rail Delivery Group (for heavy rail modes, http://data.atoc.org/data-download) and from the Bus Open Data Service (for intra-city services, <https://data.bus-data.dft.gov.uk/timetable/download/>). The maximum duration of a journey was limited to 120 minutes, departing on 22 November 2021 at 7.00 am, which represents a usual business day at the morning peak. Third, the travel duration of driving and public transport is combined into the weighted-mean travel duration, using the regional-specific mode share as weights. The weighted travel duration serves as the input to computing accessibility to vaccination.

## Appendix 2: GWR and MGWR

GWR uses an adaptive kernel to borrow a subset of data from nearby locations in order to estimate the local parameters of at any location. Therefore, it allows the coefficients of each independent variable to vary across the space, thus capturing spatial heterogeneity. GWR has been used extensively to describe spatially varying relationship between social determinants and public health topics, such as nutritional epidemiology [4], and infectious disease epidemiology [5].

The GWR model can be formulated as:

|  | $y_{i}=\beta_{i0}+\sum_{j=1}^{M} \beta_{ij}X_{ij}+\varepsilon_{i}$ | (1) |
| --- | --- | --- |

where (in the context of this study) $y_{i}$ is the percentage of the vaccinated residents in the i-th MSOA; $\beta_{i0}$ is the intercept term for the i-th MSOA; $\beta_{ij}$ is the estimated coefficient for the j-th variable in the i-th MSOA; $X_{ij}$ is the j-th variable at the i-th MSOA; $\varepsilon_{i}$ is the error term; *M* is the number of independent variable.

Whilst GWR incorporates spatial heterogeneity, it suffers from several limitations. First, this method assumes and adopts a uniform spatial bandwidth for all independent variables. That is, GWR ignores the possibility that different socio-economic variables affect the COVID-19 vaccination rates at different spatial scales, which is likely to bias the model result and limit the model performance. In addition, the local multicollinearity can lead to instability of the local parameter estimates. The formula of MGWR is similar to GWR, except that each independent variable has different bandwidths, which affects the inference of $\beta_{ij}$ coefficients.

## Appendix 3: E2SFCA

The E2SFCA was introduced by Luo and Qi [6] in order to compute accessibility to healthcare accessibility, which account for supply-to-demand ratios, the population size in the service area, and the distance decay effects. This method consists of two steps:

The first step assigns a supply-to-demand ratio to each supply point as a measure of supply availability. The numerator is the supply at location *j*, and the denominator is the sum of the demand in the catchment of location *j*. The equation is:

|  | $R_{j}=\frac{S_{j}}{\sum_{i} D_{i}w(d_{ij})}$ | (2) |
| --- | --- | --- |

where $R_{j}$ and $S_{j}$ are the supply-to-demand ratio and the capacity at supply point *j*, respectively, $D_{i}$ is the size of demand at point *i*, $d_{ij}$ is the weighted travel time between demand *i* and supply *j*,$w(d_{ij})$ is the distance decay function of $d_{ij}$. This function can take different forms, such as a step function, a negative exponential, or a Gaussian function. Herein, this function is defined as a Gaussian function that has a bandwidth parameter $d_{b}$. The Gaussian function declines slowly first and then sharply as the distance increases. In this study, the bandwidth parameter is set to 30 minutes.

The second step computes an accessibility measure at demand point *i* ($A_{i}$) by summing up the supply-to-demand ratio of supply points within the catchment area of *I*, considering the distance decay effect. The equation is:

|  | $A_{i}=\sum_{j} R_{j}w(d_{ij})$ | (3) |
| --- | --- | --- |

References

1. OSRM Project. Open Source Routing Machine. Modern C++ Routing Engine for Shortest Paths in Road Networks [Internet]. 2021 [cited 2021 Jul 23]. Available from: http://project-osrm.org/

2. Pereira RHM, Saraiva M, Herszenhut D, Braga CKV, Conway MW. r5r: Rapid Realistic Routing on Multimodal Transport Networks with R5 in R. Findings [Internet]. 2021 [cited 2021 Mar 4]; Available from: https://findingspress.org/article/21262-r5r-rapid-realistic-routing-on-multimodal-transport-networks-with-r-5-in-r

3. R Core Team. R: A language and environment for statistical computing. Vienna, Austria; 2015.

4. Yoo D. Height and death in the Antebellum United States: A view through the lens of geographically weighted regression. Econ Hum Biol. North-Holland; 2012;10:43–53.

5. Liu Y, Jiang S, Liu Y, Wang R, Li X, Yuan Z, et al. Spatial epidemiology and spatial ecology study of worldwide drug-resistant tuberculosis. Int J Health Geogr. BioMed Central; 2011;10:1–10.

6. Luo W, Qi Y. An enhanced two-step floating catchment area (E2SFCA) method for measuring spatial accessibility to primary care physicians. Health Place [Internet]. Elsevier; 2009;15:1100–7. Available from: http://dx.doi.org/10.1016/j.healthplace.2009.06.002
